# Spatiotemporal Patterns and Historical Overview of *Aedes* Mosquitoes in Iran: A Systematic Review

**DOI:** 10.3390/tropicalmed11050131

**Published:** 2026-05-12

**Authors:** Jalil Nejati, Abedin Saghafipour, Mahsa Sarvi, Rubén Bueno-Marí

**Affiliations:** 1 Health Promotion Research Center, Zahedan University of Medical Sciences, Zahedan 98167-43463, Iran; jalilnejati@yahoo.com; 2 Department of Public Health, Faculty of Health, Qom University of Medical Sciences, Qom 37169-87366, Iran; 3 Department of Public Health, School of Health, Hamadan University of Medical Sciences, Hamadan 65178-38736, Iran; mahsasarvy@gmail.com; 4 European Center of Excellence for Vector Control, Laboratorios Lokímica—Rentokil Initial, 46980 Valencia, Spain; ruben.bueno@uv.es; 5 Parasites & Health Group, Department of Pharmacy, Pharmaceutical Technology and Parasitology, Faculty of Pharmacy, University of Valencia, 46100 Valencia, Spain

**Keywords:** *Aedes*, distribution, spatial, temporal, Iran

## Abstract

*Aedes* mosquitoes are among the most important vectors of arboviral diseases such as dengue, Zika, and chikungunya. Mapping their geographic and temporal patterns is essential for understanding disease risk and guiding vector control. This systematic review provides an updated synthesis of the spatial and temporal distribution of *Aedes* species across Iran. A comprehensive search of international (PubMed, Scopus, Web of Science) and national (SID, IranMedex, Magiran) databases was performed for studies published between 1980 and 2025. Eligible publications reporting the occurrence or distribution of *Aedes* mosquitoes were screened according to PRISMA guidelines. Data were extracted and analyzed descriptively to identify long-term spatial and temporal trends. Sixty-six studies met the inclusion criteria, covering more than 20 provinces and examining over 390,000 mosquito specimens. *Aedes caspius* was the dominant species nationwide, reflecting its high ecological adaptability. Invasive vectors, *Ae. aegypti* and *Ae. albopictus*, were recorded mainly in southern coastal provinces and, more recently, in the humid northern regions. Over time, surveys have evolved from scattered faunistic reports to systematic nationwide monitoring, revealing clear patterns of ecological expansion driven by climatic and environmental factors. Increasing reports, broader geographic distribution, and adaptability to diverse ecological settings indicate an ongoing expansion of *Aedes* mosquitoes in Iran. While these developments reflect successful entomological surveillance and public health efforts, enhanced preparedness and continuous monitoring are essential to manage potential *Aedes*-borne outbreaks effectively.

## 1. Introduction

Mosquitoes (Diptera: Culicidae) are among the most important arthropods in medical and veterinary entomology due to their biting nuisance and role as vectors of numerous pathogens, such as *Plasmodium* protozoa, arboviruses, and filarial nematodes. The family Culicidae currently comprises 3727 species distributed in 41 genera, which are classified into two subfamilies, Anophelinae (three genera) and Culicinae (38 genera grouped into 11 tribes) [1]. In Culicinae, the genera *Aedes*, *Culex*, and *Anopheles* are of greatest medical significance. To date, over 950 species of *Aedes*, approximately 817 species of *Culex*, and around 500 species of *Anopheles* have been described worldwide [2,3,4].

Within the subfamily Culicinae, particular attention has been directed toward members of the tribe Aedini, which includes the genus *Aedes* Meigen, 1818. This genus includes species of high epidemiological relevance, such as *Aedes aegypti* and *Ae. albopictus*, both globally recognized as vectors of arboviral diseases including dengue, chikungunya, and Zika. Some species of this genus are considered important vectors of yellow fever, West Nile fever, Japanese encephalitis, St. Louis encephalitis, Rift Valley fever, and Sindbis fever [5]. The taxonomic placement of *Aedes* species has also been a subject of debate among specialists. Historically, many species now placed in *Aedes* were previously classified under the genus *Ochlerotatus* Lynch Arribálzaga, 1891, but were reclassified following the morphological and phylogenetic analyses conducted by Reinert et al. (2004) [6].

In Iran, 73 mosquito species belonging to the family Culicidae have been documented to date. Among them, 15 species are classified within the genus *Aedes* Meigen, 1818, representing six subgenera including *Aedes* Meigen, 1818; *Aedimorphus* Theobald, 1903; *Dahliana* Reinert, Harbach & Kitching, 2006; *Fredwardsius* Reinert, 2000; *Ochlerotatus* Lynch Arribálzaga, 1891; and *Stegomyia* Theobald, 1901 [1]. In recent years, the reporting of new *Aedes* species such as *Aedes* (*Aedes*) *cinereus* Meigen, 1818, together with the re-emergence of *Ae. aegypti* in southern Iran and the new appearance of *Ae. albopictus* in northern regions, has not only renewed scientific attention toward this genus but also raised significant public health concerns. Following the establishment of *Ae. aegypti* in southern and southeastern provinces, indigenous dengue cases have been reported, underscoring the potential for future outbreaks [1,7,8].

To date, no comprehensive systematic review has examined the spatial and temporal distribution of *Aedes* mosquito species across Iran. This review, unlike previous regional or species-specific surveys, provides the first nationwide synthesis, covering more than four decades of historical records alongside recent surveillance data. By doing so, it clarifies long-term spatiotemporal trends, highlights recent invasion events, and discusses their implications for public health preparedness. Summarizing the available evidence on *Aedes* distribution and entomological surveys across different regions of the country can guide policymakers and public health authorities in assessing the risk of *Aedes*-borne diseases and emerging arboviral threats.

## 2. Materials and Methods

**Figure 1:**
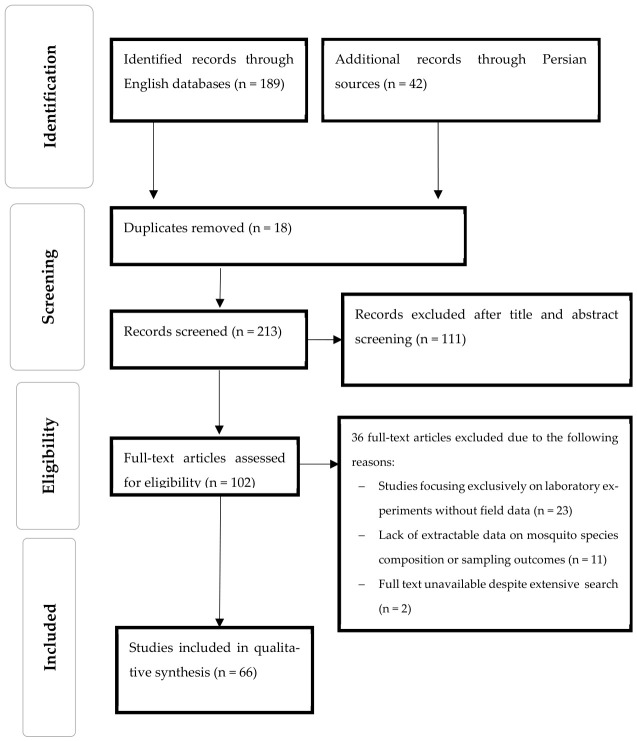
PRISMA 2020 flow diagram illustrating the study selection process for the systematic review of *Aedes* mosquito species in Iran.

This systematic review was conducted following the PRISMA 2020 guidelines [9], focusing on the spatial and temporal distribution of *Aedes* mosquitoes across Iran. The study selection process is summarized in the PRISMA 2020 flow diagram (Figure 1). A comprehensive literature search was performed across international databases, including PubMed, Scopus, Web of Science, ScienceDirect, and Google Scholar, as well as Iranian databases such as SID, IranMedex, and Magiran. The search strategy was developed based on previous reviews and employed Medical Subject Headings (MeSH) terms [10] and free-text keywords related to *Aedes* mosquitoes, including “Mosquitoes”, “Culicidae”, “*Aedes*”, and “Species”, combined with spatial distribution terms such as “Iran”, “distribution”, “presence”, and “emergence”, as well as additional terms including “surveillance” and “monitoring”. Additionally, searches were performed in Persian within Iranian databases using equivalent local terms to ensure comprehensive coverage of national studies. The full electronic search strategy, including all MeSH terms and keywords, is provided in Supplementary File S1.

This review included all descriptive, cross-sectional, and interventional investigations addressing mosquito fauna in Iran, with particular emphasis on studies reporting the collection of *Aedes* species. Only publications written in Persian or English and published between January 1980 and October 2025 met the inclusion criteria. Eligible studies were required to include information on mosquito collection (adult or larval stages), sampling locations, and sample size.

Studies were excluded if they lacked sufficient information on mosquito sampling or distribution, were review articles, letters to the editor, notes, conference abstracts, or meta-analyses. Duplicates and studies without full texts were also excluded. However, studies reporting the absence of *Aedes* species within collected Culicidae populations were included if they met the defined inclusion criteria.

All obtained articles were imported into EndNote, and duplicates were removed. Titles and abstracts were independently screened by two researchers to exclude irrelevant studies. Subsequently, the full texts of eligible articles were assessed, and relevant data were extracted. Any disagreements were resolved through consultation with a third researcher to ensure consistency. Data were collected using a pre-designed Excel form and included details such as the detected *Aedes* species, study location, total sample size, year of publication, and the life stage of the specimens (larval or adult). Finally, the corresponding maps were generated using ArcGIS software (ver.10.8).

Given the descriptive nature of most included studies, a formal risk of bias assessment tool was not applied. However, potential sources of bias were considered, including heterogeneity in sampling methods (e.g., larval surveys versus adult trapping), variation in sampling effort across provinces and time periods, and differences in species identification techniques, particularly in older studies lacking molecular confirmation. These factors may influence the apparent distribution and frequency of *Aedes* species and should be considered when interpreting the results.

Ethical approval for this study was obtained from the Ethics Committee of Qom University of Medical Sciences (IR.MUQ.REC.1403.245).

## 3. Results

A total of 66 studies were included in this systematic review. These studies were conducted across various regions of Iran, covering more than 20 provinces, and involved surveys of both larval and adult mosquitoes. Overall, over 390,000 adult and larval mosquito specimens were examined, with members of the genus *Aedes* being identified in most of the surveyed provinces.

The most frequently reported species were *Ae. caspius* (reported in more than 80% of the studies), followed by *Ae. vexans*, *Ae. geniculatus*, *Ae. caballus*, *Ae. vittatus*, and *Ae. echinus*. In recent years, the presence of *Ae. aegypti* and *Ae. albopictus*, known vectors of dengue, Zika, and chikungunya, have been confirmed mainly in the southern, southeastern, and northern provinces.

A synthesis of *Aedes* species recorded in Iran, including their status, major regions, habitats, and reporting timeline, is presented in Table 1. Detailed survey data are provided in Supplementary Table S1.

Some entomological surveys, including larval and/or adult mosquito sampling, conducted in several regions of Iran have reported the absence of *Aedes* mosquitoes. These surveys include investigations carried out in selected areas of southern, western, and even northern Iran, such as parts of Fars, Isfahan, Ilam, Lorestan, Kurdistan, and Mazandaran provinces [11,12,13,14,15,16,17,18,19,20].

**Table 1:** Synthesis of *Aedes* species recorded in Iran, their geographic distribution, ecological characteristics, and reporting timeline.

*Aedes* Species	Status	Main Geographic Region/s in Iran	Key Habitats	First Report	Most Recent Reports	Ref. No.
*Ae. aegypti*	Invasive	South, Southeast	Artificial containers; more in urban and peri-urban areas	Early 20th century 1977 (historical); re-detected 2017	2022–2024	[8,21,22,23,24]
*Ae. albopictus*	Invasive	North (Caspian region); sporadic records in South	Artificial containers; Vegetated environments; more in rural areas	2016	2024–2025	[20,25,26,27,28]
*Ae. caballus*	Native	South, Southeast	Temporary water bodies; floodplains, marshes; rural and semi-rural areas	1984	2024	[20,22,23,26,29,30,31,32,33,34,35]
*Ae. cinereus*	Native (Sporadic)	North (Caspian region), mountainous forested areas)	Temporary woodland pools; shaded rain-filled depressions; forest margins	2024	2024	[36]
*Ae. caspius*	Native	Widespread (all regions with suitable wetlands)	Permanent or temporary water bodies; marshes; rain pools; mostly fresh and less brackish water	1984	Frequently reported (2002–2025)	[7,20,22,23,25,26,27,29,30,31,34,35,37,38,39,40,41,42,43,44,45,46,47,48,49,50,51,52,53,54,55,56,57,58,59,60,61,62]
*Ae. detritus*	Native	South, Southeast	Coastal salt marshes; brackish wetlands; saline temporary water bodies	1984	2016–2017	[20,25,34,35,63]
*Ae. echinus*	Native	North	Water-filled tree holes	1984	Sporadically reported (2002–2023)	[7,34,35,37,51,64,65]
*Ae. flavescens*	Native	Rare and localized records in Southeast and Northwest	Temporary and vegetated pools typically in meadow	1984	Sporadically reported (2016–2018)	[20,26,34,56,57]
*Ae. geniculatus*	Native	North (Caspian region); localized forested areas	Water-filled tree holes	1984	Sporadically reported (2017–2023)	[7,34,35,37,46,51,64,65,66,67,68]
*Ae. leucomelas*	Native (rare)	Rare records from South	Vegetated pools in sunlit streambeds	1984	Rarely reported (2023)	[34,35,63]
*Ae. pulchritarsis*	Native (rare)	North (Caspian region)	Temporary natural pools; rain-filled ground	1984	Rarely reported (2022)	[7,34,65,67]
*Ae. vexans*	Native	Widespread (most regions with suitable floodplains)	Temporary floodwater and standing fresh water bodies; typically in rural and semi-rural environments	1984	Frequently reported (2002–2025)	[7,20,22,23,25,26,29,30,31,34,35,37,39,47,49,53,56,57,58,61,64,65,69]
*Ae. vittatus*	Native (rare/sporadic records)	South, Southeast, and Southwest	Small, sun-exposed and clear freshwater habitats in natural or semi-natural environments	1984	Rarely reported (2014–2023)	[20,22,23,34,35,45,63]
*Ae. unilineatus*	Native (rare record)	Southeast	Temporary water bodies; rural and semi-rural areas	2017	Rarely reported (single record, 2017)	[25]

### 3.1. Temporal Trend

Over the past four decades, research on *Aedes* mosquitoes in Iran have followed a distinct temporal trajectory, which can be divided into three main phases.

#### 3.1.1. Phase I (1984–2005)

During this early period, mosquito surveys were sporadic and geographically limited. Most studies focused on faunistic descriptions, identifying *Ae. caspius* and *Ae. vexans* as the dominant species. Records were mainly concentrated in northern and southwestern provinces, reflecting the limited capacity for entomological monitoring and surveillance at that time.

#### 3.1.2. Phase II (2006–2015)

This decade marked a gradual expansion of entomological studies into new ecological zones, accompanied by improvements in mosquito taxonomy and surveillance. Additional species such as *Ae. geniculatus*, *Ae. caballus*, *Ae. vittatus*, and *Ae. echinus* were recorded from different regions. Increased field activities in northern, central, and southern Iran led to the detection of *Aedes* mosquitoes in previously uninvestigated habitats, including wetlands and peri-urban areas.

#### 3.1.3. Phase III (2016–2025)

A notable surge in *Aedes*-related research occurred during this period, coinciding with the reappearance of *Stegomyia* mosquitoes. After more than seventy years, *Ae. aegypti* was redetected in southern Iran, initially in Bandar Khamir (March 2017) [21], later in Bandar Lengeh (December 2019) [21], and subsequently in Bandar Abbas [8], suggesting re-establishment and potential local adaptation. The detection of *Ae. albopictus* in Sistan and Baluchestan (2016) [20] and later in Guilan (2023) [28] demonstrated its ability to survive in both the humid southern and northern regions of Iran.

### 3.2. Spatial Distribution

**Figure 2:**
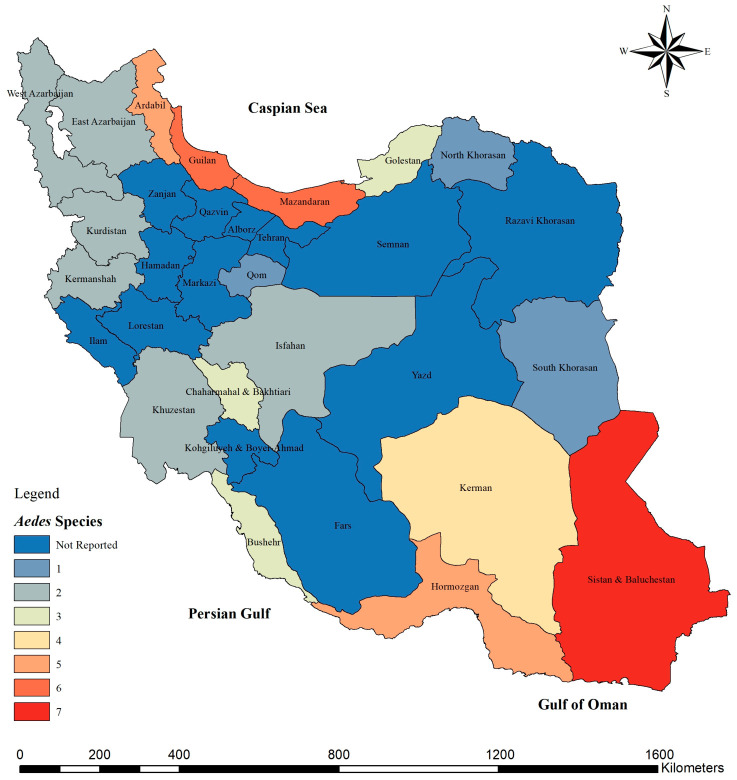
Spatial distribution and species diversity of *Aedes* mosquitoes across Iran, showing the number of recorded species in each province.

**Figure 3:**
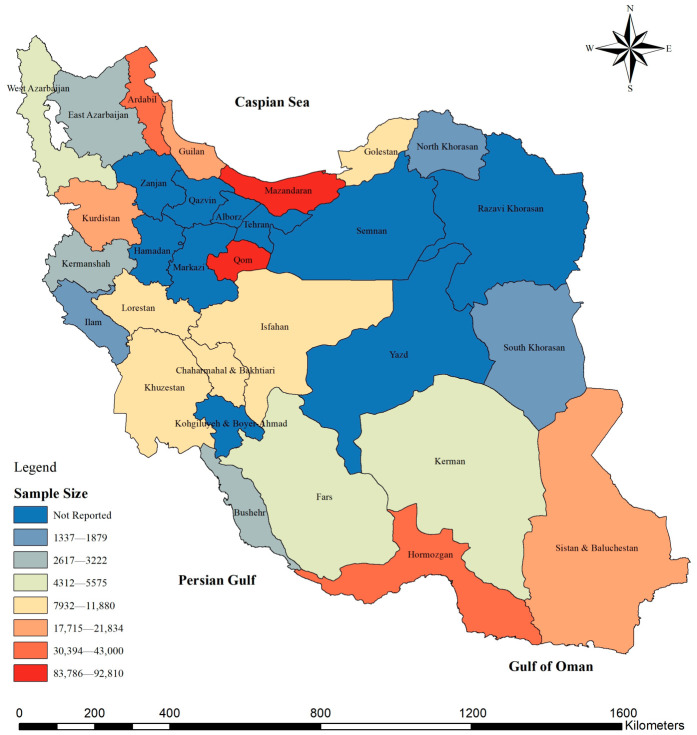
Spatial distribution of total mosquito sample sizes (larval and adult stages) reported in *Aedes* studies across Iranian provinces. Color intensity represents increasing sample size.

The spatial distribution of *Aedes* mosquitoes in Iran shows a distinct pattern shaped by climatic gradients and ecological diversity. Overall, *Aedes* species have been reported from more than 20 provinces, with the highest diversity and abundance observed along the northern Caspian coast and the southern coastal regions of the Persian Gulf and Oman Sea (Figure 2). Similarly, the total mosquito sample size followed the same pattern, being largely concentrated in the northern and southern coastal provinces (Figure 3).

#### 3.2.1. Northern Provinces (Caspian Region)

The provinces of Guilan, Mazandaran, and Golestan provide stable habitats for several *Aedes* species, including *Ae. caspius*, *Ae. vexans*, *Ae. geniculatus*, *Ae. pulcritarsis*, and *Ae. echinus*. The humid subtropical climate, permanent wetlands, rice paddies, and dense vegetation offer favorable breeding environments throughout most of the year. Recent detections of *Ae. albopictus* in several counties of Guilan and subsequently Mazandaran [1,28], indicate both suitable climatic conditions and ongoing northern expansion along the Caspian Sea coast.

#### 3.2.2. Central and Western Regions

In provinces such as Qom, Isfahan, and Kurdistan, *Aedes* diversity is comparatively lower, dominated by *Ae. caspius* and *Ae. vexans*. In Qom, exceptionally high adult densities were recorded in 2025, demonstrating the adaptability of *Ae. caspius* to arid and semi-arid habitats. In contrast, mountainous and cooler regions such as Kurdistan and Lorestan yielded no *Aedes* specimens in several surveys, possibly due to climatic limitations and scarcity of suitable larval habitats.

#### 3.2.3. Southern and Southeastern Provinces

The southern coastal belt, including Hormozgan, Bushehr, Khuzestan, and Sistan and Baluchestan, represents the most dynamic area for *Aedes* distribution. These regions host both native and invasive species. *Aedes caspius*, *Ae. vittatus*, *Ae. caballus*, and *Ae. detritus* are widely distributed, while *Ae. aegypti* and *Ae. albopictus* have emerged and gradually expanded in recent years. The stepwise *detection* of *Ae. aegypti* from Bandar Khamir (2017) [21] to Chabahar (2023) [70] illustrates a clear southeastward expansion along the Persian Gulf and Oman Sea coasts.

## 4. Discussion

The findings of this systematic review present a comprehensive and up-to-date picture of species composition, distribution, and recent changes in *Aedes* mosquitoes in Iran. Over the past four decades, the country has shown marked variations in the presence of *Aedes* species over time and regions, likely influenced by ecological, climatic, and human-related factors.

The marked rise in *Aedes* reports after 2015 appears to be linked to strengthened vector surveillance programs. Following the first report of the invasive species *Ae. albopictus* in southeastern Iran in 2015, which coincided with dengue outbreaks in Pakistan, Iran’s eastern neighbor, concerns within the health system increased. This was mainly because the potential occurrence of dengue outbreaks alongside ongoing malaria transmission could further intensify existing challenges in the health sector. Therefore, strengthening entomological surveillance and vector monitoring was prioritized. As part of this new operational approach, the research focus also shifted, and numerous studies were initiated in different regions of the country to monitor the presence of dengue vectors. Because these studies monitored a wide range of larval habitats, an increase in both the number of reports and the diversity of recorded *Aedes* species was observed.

A modelling study in Iran predicted that climatic shifts, particularly in coastal zones, will increase habitat suitability for *Ae. aegypti* and *Ae. albopictus* in the coming decades [10,71]. In other words, the rising number of reports of *Aedes* mosquitoes in Iran over the past decade cannot be explained solely by enhanced surveillance efforts. Instead, this trend appears to reflect a complex interaction between climatic, environmental, and human factors. Similar patterns have also been reported in other countries such as Pakistan and Saudi Arabia, where increased surveillance and climate variability have been associated with greater *Aedes* abundance and broader distribution [72,73]. The gradual rise in mean annual temperatures and the extension of warm seasons, especially in the southern and northern coastal areas of Iran, may enhance larval development, adult survival, and activity, and ultimately the vectorial capacity of *Ae. aegypti* and *Ae. albopictus* [41]. Concurrently, irregular rainfall patterns and events such as urban flooding can create temporary breeding sites, thereby facilitating oviposition and the expansion of local transmission foci [74]. In addition to these climatic drivers, anthropogenic changes play a key role. Urbanization, the growth of informal settlements, and shifts in household water-storage practices in water-limited areas have increased the availability of artificial larval habitats, conditions that are particularly favorable for *Stegomyia* species such as *Ae. aegypti* [75]. These findings suggest that regional climatic differences do not act as barriers; instead, through the ecological adaptability of *Aedes* mosquitoes, they function as facilitating factors for their geographical expansion. Collectively, these observations emphasize the dynamic nature of *Aedes* distribution in Iran and highlight the importance of continuous vector surveillance in both endemic and at-risk areas.

Based on our results, humid and semi-humid climates, such as the northern (Caspian) and southern (Persian Gulf and Oman Sea) provinces of Iran, harbor the highest diversity and abundance of *Aedes* mosquitoes. In the north, provinces such as Guilan, Mazandaran, and Golestan contain numerous permanent larval habitats, wetlands, rice paddies, and forested zones, which support stable *Aedes* populations [12]. The southern provinces with humid climates, including Hormozgan, Bushehr, Khuzestan, and Sistan and Baluchestan, also host both native and invasive species [31,61,73]. Global distribution models of *Ae. aegypti* and *Ae. albopictus* indicate that tropical and subtropical regions with sustained humidity and moderate temperatures remain their primary ecological range. Supporting this, a recent study in Central Africa found that rainfall and humidity were significant predictors of *Aedes* abundance, suggesting that moist environments play a key role in mosquito population increase [76].

*Aedes caspius* was the dominant species across almost all ecological settings, reflecting remarkable adaptability to diverse environments, ranging from permanent to temporary and fresh to brackish water bodies. Its ability to adapt to changing conditions likely explains its wide distribution and persistence across different seasons and environments. This observation aligns with studies from Mediterranean and North African regions, where *Ae. caspius* similarly exhibits high tolerance to variable salinity, temperature, and habitat types. In contrast, species such as *Ae. caballus*, *Ae. vittatus*, *Ae. echinus*, and *Ae. geniculatus* display more localized distributions, each specialized for specific habitats [33,37]. While their habitat specialization may limit population density, it also highlights their potential importance in sustaining localized arboviral transmission cycles.

The most notable outcome of this review is the re-emergence of invasive *Stegomyia* mosquitoes in Iran after an absence of more than seven decades. *Aedes aegypti*, the primary vector of dengue, Zika, and chikungunya, was historically documented in Khorramshahr and Bushehr during the early 20th century but was not recorded again until 2017 [5]. Its subsequent spread from Bandar Khamir to Chabahar between 2017 and 2023 demonstrates a progressive re-establishment along southern coastal regions, highlighting the potential risk for arboviral outbreaks in these densely populated areas [21,70]. Importantly, the presence of invasive *Aedes* species in southern Iran coincides with recent disease notification data, particularly indigenous dengue cases reported from provinces such as Hormozgan and Sistan and Baluchestan. The detection and apparent establishment of *Ae. aegypti* in these regions, together with documented dengue transmission, underscore a real risk for sustained arboviral circulation [77,78]. This overlap between vector presence and disease occurrence highlights the public health relevance of the observed spatiotemporal patterns and emphasizes the need for integrated entomological and epidemiological surveillance.

*Aedes albopictus*, although first reported in southern Iran in 2016, was not confirmed to establish stable populations in those areas. Its recent detection in northern provinces, including Guilan and Mazandaran, suggests adaptation to more humid and temperate conditions at higher latitudes [28]. This northern expansion has not yet been accompanied by reported autochthonous dengue cases; however, its establishment in climatically suitable areas suggests a potential future risk that warrants proactive monitoring.

The invasion and establishment patterns of *Ae. aegypti* and *Ae. albopictus* in Iran closely resemble those reported from the Mediterranean basin and southern Europe, where vector introduction has frequently occurred through ports via used tires, shipping containers, and commercial cargo, followed by gradual spread into urban and peri-urban settings. In this context, previous studies consistently highlight international trade as one of the major drivers of the global expansion of *Ae. aegypti* and *Ae. albopictus* [74]. Experiences from Pakistan and Saudi Arabia further demonstrate that overlooking cross-border movements and delaying targeted interventions can facilitate a rapid transition from vector presence to the emergence of indigenous epidemics [77]. From this perspective, Iran and many Mediterranean countries are currently at a critical stage in which active surveillance, early control measures, and the application of regional lessons learned may prevent the establishment of sustained transmission cycles [79]. Collectively, this evidence reinforces the need for flexible, region-specific, and evidence-based strategies.

The spatiotemporal evidence synthesized in this review suggests that *Aedes* surveillance and control strategies in Iran should be targeted, region-specific, and risk-based. In southern and southeastern provinces, where *Ae. aegypti* has become established and coincides with autochthonous dengue cases, sustained vector surveillance, evaluation of control effectiveness, and the integration of entomological and epidemiological data may substantially strengthen early warning capacities [70,77]. By contrast, in northern regions where climatic conditions are progressively becoming more favorable for *Ae. albopictus*, priority should be given to larval source management, removal of small urban breeding sites, and preventive interventions before stable vector populations are established. Experimental evidence indicates that reducing immature mosquito densities not only constrains adult populations but may also alter interspecific competition and even adult susceptibility to viral infections [80]. These considerations underscore that control strategies should move beyond reactive responses and instead focus on prevention and early intervention. Furthermore, an exclusive emphasis on traditional domestic habitats risks underestimating the contribution of non-residential environments. Recent studies demonstrate that workshops, storage facilities, and semi-industrial spaces can play a significant role in egg production and the persistence of *Aedes* populations, habitats that are often inadequately addressed in urban and peri-urban control programs [75]. Collectively, these findings emphasize the need to reassess surveillance and control strategies in accordance with the growing complexity of contemporary habitat patterns.

The observed spatial patterns should be interpreted with caution due to methodological heterogeneity among the included studies. Provinces with more intensive entomological surveillance may appear to have higher *Aedes* diversity simply as a result of greater sampling effort, while the absence of records in other regions does not necessarily indicate true absence. Additionally, variations in collection methods and identification approaches across studies may have affected species detection. Acknowledging these limitations is essential for interpreting the findings and further highlights the need for standardized, continuous, and nationally coordinated *Aedes* surveillance across Iran.

## 5. Conclusions

While the increasing number of records can partly reflect improvements in entomological surveillance and research, they also indicate an expanding geographic range and high adaptability of *Aedes* mosquitoes across Iran, with possible effects on future disease transmission. In addition, the emergence of *Ae. aegypti* in southern and southeastern regions and *Ae. albopictus* in humid northern areas can reflect a complex interaction of climatic, environmental, and human factors that may influence the epidemiology of arboviral diseases in the country. These findings highlight the importance of sustained and targeted surveillance. Strengthening preparedness within the public health system, together with continuous entomological monitoring, will be essential to reduce the risk of *Aedes*-borne disease emergence and spread in Iran.

## Data Availability

The datasets used and/or analysed during the current study are available from the corresponding author on reasonable request.

## Supplementary Materials

The following supporting information can be downloaded at: https://www.mdpi.com/article/10.3390/tropicalmed11050131/s1, File S1: Electronic Search Strategy; Table S1: Detailed entomological survey data on the spatial and temporal distribution of *Aedes ‎mosquitoes* in Iran.

## References

[1] Azari-Hamidian S., Harbach R.E. (2025). Updated checklist of the mosquitoes (Diptera: Culicidae) known to occur in Iran, with updated keys to the genera, subgenera and species of *Aedes*. Zootaxa.

[2] Monteiro V.V.S., Navegantes-Lima K.C., de Lemos A.B., da Silva G.L., de Souza Gomes R., Reis J.F., Rodrigues Junior L.C., da Silva O.S., Romão P.R.T., Monteiro M.C. (2019). *Aedes*-Chikungunya Virus Interaction: Key Role of Vector Midguts Microbiota and Its Saliva in the Host Infection. Front. Microbiol..

[3] Reis L.A.M., Pampolha A.B.O., Nascimento B., Dias D.D., Araújo P., Silva F.S.D., Silva L., Reis H.C.F., Silva E., Nunes Neto J.P. (2023). Genus *Culex Linnaeus*, 1758 (Diptera: Culicidae) as an Important Potential Arbovirus Vector in Brazil: An Integrative Review. Life.

[4] Das B., Ghosal S., Mohanty S. (2018). Aedes: What Do We Know About Them and What Can They Transmit?.

[5] Azari-Hamidian S. (2007). Checklist of Iranian mosquitoes (Diptera: Culicidae). J. Vector Ecol..

[6] Reinert J.F., Harbach R.E., Kitching I.J. (2004). Phylogeny and classification of Aedini (Diptera: Culicidae), based on morphological characters of all life stages. Zool. J. Linn. Soc..

[7] Nikookar S.H., Charkame A., Nezammahalleh A., Moradi-Asl E., Enayati A., Fazeli-Dinan M., Sedaghat M.M., Zaim M. (2023). Entomological surveillance of invasive *Aedes* mosquitoes in Mazandaran Province, northern Iran from 2014 to 2020. Sci. Rep..

[8] Paksa A., Azizi K., Yousefi S., Dabaghmanesh S., Shahabi S., Sanei-Dehkordi A. (2024). First report on the molecular phylogenetics and population genetics of *Aedes aegypti* in Iran. Parasit. Vectors.

[9] Page M.J., McKenzie J.E., Bossuyt P.M., Boutron I., Hoffmann T.C., Mulrow C.D., Shamseer L., Tetzlaff J.M., Akl E.A., Brennan S.E. (2021). The PRISMA 2020 statement: An updated guideline for reporting systematic reviews. BMJ.

[10] Saeed A., Ali S., Khan F., Muhammad S., Reboita M.S., Khan A.W., Goheer M.A., Khan M.A., Kumar R., Ikram A. (2023). Modelling the impact of climate change on dengue outbreaks and future spatiotemporal shift in Pakistan. Environ. Geochem. Health.

[11] Soltani Z., Keshavarzi D., Ebrahimi M., Soltani A., Moemenbellah-Fard M.J., Soltani F., Faramarzi H., Amraee K., Elyasigomari A. (2017). The Fauna and Active Season of Mosquitoes in West of Fars Province, Southwest of Iran. Arch. Razi Inst..

[12] Nikookar S.H., Fazeli-Dinan M., Azari-Hamidian S., Mousavinasab S.N., Arabi M., Ziapour S.P., Shojaee J., Enayati A. (2017). Species composition and abundance of mosquito larvae in relation with their habitat characteristics in Mazandaran Province, northern Iran. Bull. Entomol. Res..

[13] Kayedi M.H., Sepahvand F., Mostafavi E., Chinikar S., Mokhayeri H., Chegheni Sharafi A., Wong G., Shahhosseini N., Moosa Kazemi S.H. (2020). Morphological and molecular identification of Culicidae mosquitoes (Diptera: Culicidae) in Lorestan province, Western Iran. Heliyon.

[14] Kassiri H., Dehghani R., Khodkar I., Moosakzemi S.H., Asgarian T., Golafshan A.H., Maleki F., Kazemi G. (2021). Determination of fauna and abundance of larvae of the mosquitoes (Diptera: Culicidae) in paddy fields in Lenjan and Mobarakeh Counties, Isfahan Province, Center of Iran. J. Entomol. Res..

[15] Soltani A., Hoseini Z., Azizi K., Alipour H. (2021). A faunal study on medically important mosquitoes (Diptera: Culicidae) in Qir and Karzin from Fars province, southern Iran, during 2017-18. J. Entomol. Acarol. Res..

[16] Salavati B., Zahirnia A.H., Nasirian H., Azari-Hamidian S. (2021). Trend of mosquito (Diptera: Culicidae) monthly distribution in Sanandaj County of Iran. Biodiversitas.

[17] Moosa-Kazemi S.H., Etemadi Y., Sedaghat M.M., Vatandoost H., Mokhayeri H., Kayedi M.H. (2021). Investigation on Mosquitoes Fauna (Diptera: Culicidae) and Probable Vector of West Nile Virus in Lorestan Province, Western Iran. J. Arthropod Borne Dis..

[18] Asgarian T.S., Moosa-Kazemi S.H., Sedaghat M.M., Dehghani R., Yaghoobi-Ershadi M.R. (2021). Fauna and Larval Habitat Characteristics of Mosquitoes (Diptera: Culicidae) in Kashan County, Central Iran, 2019. J. Arthropod Borne Dis..

[19] Sharifi F., Banafshi O., Rasouli A., Ghoreishi S., Saeedi S., Khalesi M., Rezai A., Moradi Asl E., Zareie B., Veisi Khodlan N. (2022). Biodiversity and Spatial Distribution of Mosquitoes (Diptera: Culicidae) in Kurdistan Province, Western Iran. J. Arthropod Borne Dis..

[20] Doosti S., Yaghoobi-Ershadi M.R., Schaffner F., Moosa-Kazemi S.H., Akbarzadeh K., Gooya M.M., Vatandoost H., Shirzadi M.R., Mosta-Favi E. (2016). Mosquito Surveillance and the First Record of the Invasive Mosquito Species *Aedes* (*Stegomyia*) *albopictus* (Skuse) (Diptera: Culicidae) in Southern Iran. Iran. J. Public Health.

[21] Dorzaban H., Soltani A., Alipour H., Hatami J., Hashemi S.A.J., Shahriari-Namadi M., Safari R., Azizi K. (2020). Morphological and molecular-based identification of *Aedes aegypti* (Diptera: Culicidae), a main vector of Dengue Fever, the first record in Iran after decades. Research Square.

[22] Dorzaban H., Soltani A., Alipour H., Hatami J., Jaberhashemi S.A., Shahriari-Namadi M., Paksa A., Safari R., Talbalaghi A., Azizi K. (2022). Mosquito surveillance and the first record of morphological and molecular-based identification of invasive species *Aedes* (*Stegomyia*) *aegypti* (Diptera: Culicidae), southern Iran. Exp. Parasitol..

[23] Azizi K., Dorzaban H., Soltani A., Alipour H., Jaberhashemi S.A., Salehi-Vaziri M., Mohammadi T., Fereydouni Z., Paksa A. (2023). Monitoring of dengue virus in field-caught *Aedes* species (Diptera: Culicidae) by molecular method, from 2016 to 2017 in Southern Iran. J. Health Sci. Surveill. Syst..

[24] Enayati A., Valadan R., Bagherzadeh M., Cheraghpour M., Nikookar S.H., Fazeli-Dinan M., Hosseini-Vasoukolaei N., Sahraei Rostami F., Shabani Kordshouli R., Raeisi A. (2024). Kdr genotyping and the first report of V410L and V1016I kdr mutations in voltage-gated sodium channel gene in *Aedes aegypti* (Diptera: Culicidae) from Iran. Parasit. Vectors.

[25] Yaghoobi-Ershadi M.R., Doosti S., Schaffner F., Moosa-Kazemi S.H., Akbarzadeh K., Yaghoobi-Ershadi N. (2017). Morphological studies on adult mosquitoes (Diptera: Culicidae) and first report of the potential Zika virus vector *Aedes* (*Stegomyia*) *unilineatus* (Theobald, 1906) in Iran. Bull. Soc. Pathol. Exot..

[26] Doosti S., Yaghoobi-Ershadi M., Sedaghat M., Akbarzadeh K., Godwin G.N. (2021). Larval habitats characteristics of Culicinae subfamily in the south of Iran. Nus. Biosci..

[27] Fakour S., Soltanalinejad P., Abishvand J., Sharifi H., Asayeshi M., Ghorbani E., Asghari Jajin S., Kamrani S., Soleymani M., Rasouli M. (2025). First Morphological and Ecological Report of *Aedes albopictus*, the Dengue Fever Vector, in Ardabil Province, Northwestern Iran. J. Ardabil Univ. Med. Sci..

[28] Azari-Hamidian S., Norouzi B., Maleki H., Rezvani S.M., Pourgholami M., Oshaghi M.A. (2024). First record of a medically important vector, the Asian tiger mosquito *Aedes albopictus* (Skuse, 1895)(Diptera: Culicidae), using morphological and molecular data in northern Iran. J. Insect Biodivers. Syst..

[29] Moosa-Kazemi S., Vatandoost H., Nikookar H., Fathian M. (2009). Culicinae (Diptera: Culicidae) mosquitoes in chabahar county, sistan and baluchistan province, southeastern Iran. Iran. J. Arthropod Borne Dis..

[30] Hanafi-Bojd A.-A., Soleimani-Ahmadi M., Doosti S., Azari-Hamidian S. (2017). Larval habitats, affinity and diversity indices of Culicinae (Diptera: Culicidae) in southern Iran. Int. J. Mosq. Res..

[31] Nejati J., Zaim M., Vatandoost H., Moosa-Kazemi S.H., Bueno-Marí R., Azari-Hamidian S., Sedaghat M.M., Hanafi-Bojd A.A., Yaghoobi-Ershadi M.R., Okati-Aliabad H. (2020). Employing Different Traps for Collection of Mosquitoes and Detection of Dengue, Chikungunya and Zika Vector, *Aedes albopictus*, in Borderline of Iran and Pakistan. J. Arthropod Borne Dis..

[32] Jaberhashemi S.A., Azari-Hamidian S., Soltani A., Azizi K., Dorzaban H., Norouzi M., Daghighi E. (2022). The Fauna, Diversity, and Bionomics of Culicinae (Diptera: Culicidae) in Hormozgan Province, Southern Iran. J. Med. Entomol..

[33] Nejati J., Azari-Hamidian S., Oshaghi M.A., Vatandoost H., White V.L., Moosa-Kazemi S.H., Bueno-Marí R., Hanafi-Bojd A.A., Endersby-Harshman N.M., Axford J.K. (2024). The monsoon-associated equine South African pointy mosquito ‘*Aedes caballus*’; the first comprehensive record from southeastern Iran with a description of ecological, morphological, and molecular aspects. PLoS ONE.

[34] Zaim M., Manouchehri A., Ershadi M. (1984). Mosquito fauna of Iran. I. *Aedes* (Diptera: Culicidae). Iran. J. Public Health.

[35] Zaim M. (1987). The distribution and larval habitat characteristics of Iranian Culicinae. J. Am. Mosq. Control Assoc..

[36] Azari-Hamidian S., Norouzi B., Maleki H., Rezvani S.M., Pourgholami M., Oshaghi M.A. (2024). First record of *Aedes* (*Aedes*) *cinereus* (Diptera: Culicidae) in Iran. Zool. Middle East..

[37] Azari-Hamidian S., Joeafshani M.A., Rassaei A.R., Mosslem M. (2003). Faunistic Studies on the Genus Culiseta (Diptera: Culicidae) in Guilan Province. J. Kerman Univ. Med. Sci..

[38] Abai M., Azari-Hamidian S., Ladonni H., Hakimi M., Mashhadi-Esmail K., Sheikhzadeh K., Kousha A., Vatandoost H. (2007). Fauna and Checklist of Mosquitoes (Diptera: Culicidae) of East Azerbaijan Province, Northwestern Iran. Iran. J. Arthropod-Borne Dis..

[39] Kazemi S.H., Karimian F., Davari B. (2010). Culicinae mosquitoes in Sanandaj county, Kurdistan province, western Iran. J. Vector Borne Dis..

[40] Azari-Hamidian S., Linton Y.-M., Ladonni H., Oshaghi M., Hanafi-Bojd A.A., Moosa-Kazemi S., Shabkhiz H., Pakari A., Harbach R. (2010). Mosquito (Diptera: Culicidae) fauna of the Iranian islands in the Persian Gulf. J. Nat. Hist..

[41] Azari Hamidian S., Abai M., Arzamani K., Bakhshi H., Karami H., Ladonni H., Harbach R. (2011). Mosquitoes (Diptera: Culicidae) of North Khorasan Province, northeastern Iran and the zoogeographic affinities of the Iranian and middle Asian mosquito fauna. J. Entomol..

[42] Navidpour S., Vazirianzadeh B., Harbach R., Jahanifard E., Moravvej S.A. (2012). The identification of culicine mosquitoes in the Shadegan wetland in southwestern Iran. J. Insect Sci..

[43] Saghafipour A., Abai M., Farzinnia B., Nafar R., Ladonni H., Azari-Hamidian S. (2012). Mosquito (Diptera: Culicidae) fauna of qom province, iran. J. Arthropod Borne Dis..

[44] Banafshi O., Abai M.R., Ladonni H., Bakhshi H., Karami H., Hamidian S.A. (2013). The fauna and ecology of mosquito larvae (Diptera: Culicidae) in western Iran. Yurk J. Zool..

[45] Nasirian H., Sadeghi S.M.T., Vazirianzadeh B., Moosa-Kazemi S.H. (2014). New record of *Aedes vittatus* and *Culiseta subochrea* (Diptera: Culicidae) and their distribution from Shadegan wetland, South Western Iran. J. Entomol. Zool. Stud..

[46] Khoshdel-Nezamiha F., Vatandoost H., Azari-Hamidian S., Bavani M.M., Dabiri F., Entezar-Mahdi R., Chavshin A.R. (2014). Fauna and Larval Habitats of Mosquitoes (Diptera: Culicidae) of West Azerbaijan Province, Northwestern Iran. J. Arthropod Borne Dis..

[47] Moosa-Kazemi S.H., Zahirnia A.H., Sharifi F., Davari B. (2015). The Fauna and Ecology of Mosquitoes (Diptera: Culicidae) in Western Iran. J. Arthropod Borne Dis..

[48] Farhadinejad R., Mousavi M., Amraee K. (2015). The species composition of mosquitoes (Diptera: Culicidae) in the Mahshahr district, Khuzestan province, southwest of Iran. Arch. Razi Inst..

[49] Ladonni H., Azari-Hamidian S., Alizadeh M., Abai M.R., Bakhshi H. (2015). The fauna, habitats, and affinity indices of mosquito larvae (Diptera: Culicidae) in Central Iran. N. West. J. Zool..

[50] Khoshdel-Nezamiha F., Vatandoost H., Oshaghi M.A., Azari-Hamidian S., Mianroodi R.A., Dabiri F., Bagheri M., Terenius O., Chavshin A.R. (2016). Molecular Characterization of Mosquitoes (Diptera: Culicidae) in Northwestern Iran by Using rDNA-ITS2. Jpn. J. Infect. Dis..

[51] Sofizadeh A., Moosa-Kazemi S.H., Dehghan H. (2017). Larval Habitats Characteristics of Mosquitoes (Diptera: Culicidae) in North-East of Iran. J. Arthropod Borne Dis..

[52] Sofizadeh A., Eftekhari B., Pesaraklo A.R., Mohammadnia A., Ajam F., Farrokhi Balajadeh M., Gharavi M.Y., Sanad Gol N. (2018). Species diversity and larval habitat characteristics of mosquitoes (Diptera: Culicidae) in Golestan province, 2016. Jorjani Biomed. J..

[53] Nikookar S.H., Fazeli-Dinan M., Azari-Hamidian S., Nasab S.N.M., Aarabi M., Ziapour S.P., Enayati A., Hemingway J. (2018). Fauna, Ecological Characteristics, and Checklist of the Mosquitoes in Mazandaran Province, Northern Iran. J. Med. Entomol..

[54] Doosti S., Yaghoobi-Ershadi M.R., Sedaghat M.M., Moosa-Kazemi S.H., Akbarzadeh K., Hashemi-Aghdam S.S. (2018). Genetic Population Diversity of *Aedes caspius* in Southern Provinces of Iran. Bull. Soc. Pathol. Exot..

[55] Sofizadeh A., Shoraka H.R., Mesgarian F., Ozbaki G.M., Gharaninia A., Sahneh E., Dankoob R., Malaka A., Fallah S., Nemani S. (2018). Fauna and Larval Habitats Characteristics of Mosquitoes (Diptera: Culicidae) in Golestan Province, Northeast of Iran, 2014–2015. J. Arthropod Borne Dis..

[56] Moradi-Asl E., Vatandoost H., Adham D., Emdadi D., Moosa-Kazemi H. (2019). Investigation on the Occurrence of *Aedes* Species in Borderline of Iran and Azerbaijan for Control of Arboviral Diseases. J. Arthropod Borne Dis..

[57] Moradi-Asl E., Hazrati S., Vatandoost H., Emdadi D., Ghorbani E., Ghasemian A., Rafiee M., Panahi A., Shokri A. (2018). Fauna and larval habitat characteristics of mosquitoes (Diptera: Culicidae) in Ardabil province, Northwestern Iran. J. Health.

[58] Paksa A., Sedaghat M.M., Vatandoost H., Yaghoobi-Ershadi M.R., Moosa-Kazemi S.H., Hazratian T., Sanei-Dehkordi A., Oshaghi M.A. (2019). Biodiversity of Mosquitoes (Diptera: Culicidae) with Emphasis on Potential Arbovirus Vectors in East Azerbaijan Province, Northwestern Iran. J. Arthropod Borne Dis..

[59] Amini M., Hanafi-Bojd A.A., Aghapour A.A., Chavshin A.R. (2020). Larval habitats and species diversity of mosquitoes (Diptera: Culicidae) in West Azerbaijan Province, Northwestern Iran. BMC Ecol..

[60] Hassandoust S., Moosa-Kazemi S.H., Vatandoost H., Sedaghat M.M., Akbarzadeh K. (2020). Evaluation of Susceptibility of Aedes caspius (Diptera: Culicidae) to Insecticides in a Potent Arboviral-Prone Area, Southern Iran. J. Arthropod Borne Dis..

[61] Poudat A., Edalat H., Zaim M., Rezaei F., Salim Abadi Y., Basseri H.R. (2023). Species Composition and Geographic Distribution of Culicinae Mosquitoes and Their Possible Infection with West Nile Virus in Hormozgan Province, Southern Iran. Iran. J. Public Health.

[62] Abedi-Astaneh F., Rad H.R., Izanlou H., Hosseinalipour S.A., Hamta A., Eshaghieh M., Ebrahimi M., Ansari-Cheshmeh M.A., Pouriayevali M.H., Salehi-Vaziri M. (2025). Extensive surveillance of mosquitoes and molecular investigation of arboviruses in Central Iran. Ann. Med. Surg..

[63] Paksa A., Vahedi M., Yousefi S., Saberi N., Rahimi S., Amin M. (2023). Biodiversity of mosquitoes (Diptera: Culicidae), vectors of important arboviral diseases at different altitudes in the central part of Iran. Turk. J. Zool..

[64] Azari-Hamidian S. (2011). Larval habitat characteristics of the genus *Anopheles* (Diptera: Culicidae) and a checklist of mosquitoes in guilan province, northern iran. Iran. J. Arthropod Borne Dis..

[65] Nikookar S.H., Maleki A., Fazeli-Dinan M., Shabani Kordshouli R., Enayati A. (2022). Entomological Surveillance of the Invasive *Aedes* Species at Higher-Priority Entry Points in Northern Iran: Exploratory Report on a Field Study. JMIR Public Health Surveill..

[66] Nikookar S.H., Moosa-Kazemi S.H., Yaghoobi-Ershadi M.R., Vatandoost H., Oshaghi M.A., Ataei A., Anjamrooz M. (2015). Fauna and Larval Habitat Characteristics of Mosquitoes in Neka County, Northern Iran. J. Arthropod Borne Dis..

[67] Azari-Hamidian S., Norouzi B., Noorallahi A., Hanafi-Bojd A.A. (2018). Seasonal activity of adult mosquitoes (Diptera: Culicidae) in a focus of dirofilariasis and West Nile infection in northern Iran. J. Arthropod Borne Dis..

[68] Azari-Hamidian S., Yaghoobi-Ershadi M., Javadian E., Abai M., Mobedi I., Linton Y.M., Harbach R. (2009). Distribution and ecology of mosquitoes in a focus of dirofilariasis in northwestern Iran, with the first finding of filarial larvae in naturally infected local mosquitoes. Med. Vet. Entomol..

[69] Moin-Vaziri V., Charrel R.N., Badakhshan M., de Lamballerie X., Rahbarian N., Bavani M.M., Azari-Hamidian S. (2019). A Molecular Screening of Mosquitoes (Diptera: Culicidae) for Flaviviruses in a Focus of West Nile Virus Infection in Northern Iran. J. Arthropod Borne Dis..

[70] Sedaghat M., Enayati A., Nejati J. (2025). A Changing Scenario at a Regional Gateway: New Emergence of the Invasive Dengue Vector *Aedes aegypti*, in Zahedan, Southeastern Iran. Health Scope.

[71] Nejati J., Bueno-Marí R., Collantes F., Hanafi-Bojd A.A., Vatandoost H., Charrahy Z., Tabatabaei S.M., Yaghoobi-Ershadi M.R., Hasanzehi A., Shirzadi M.R. (2017). Potential Risk Areas of *Aedes albopictus* in South-Eastern Iran: A Vector of Dengue Fever, Zika, and Chikungunya. Front. Microbiol..

[72] Al Azab A., Zaituon A., Alghamdi K., Abd Al Galil F. (2022). Surveillance of Dengue Fever Vector *Aedes aegypti* in Different Areas in Jeddah City Saudi Arabia. Adv. Anim. Vet. Sci..

[73] Nejati J., Bueno-Marí R. (2024). Malaria and dengue outbreaks: A double health threat in southeastern Iran. J. Vector Borne Dis..

[74] Padonou G.G., Konkon A.K., Salako A.S., Zoungbédji D.M., Osse R., Sovi A., Azondekon R., Sidick A., Ahouandjinou J.M., Adoha C.J. (2023). Distribution and Abundance of *Aedes aegypti* and *Aedes albopictus* (Diptera: Culicidae) in Benin, West Africa. Trop. Med. Infect. Dis..

[75] Soto-López J.D., Barrios-Izás M.A., Vieira Lista M.C., Muro A. (2024). Role of Non-Residential Larval Habitats in *Aedes* Spatiotemporal Egg Production. Life.

[76] Montgomery M.J., Harwood J.F., Yougang A.P., Wilson-Bahun T.A., Tedjou A.N., Keumeni C.R., Wondji C.S., Kamgang B., Kilpatrick A.M. (2025). The effects of urbanization, temperature, and rainfall on *Aedes aegypti* and *Aedes albopictus* mosquito abundance across a broad latitudinal gradient in Central Africa. Parasit. Vectors.

[77] Nikookar S.H., Hoseini S., Dehghan O., Fazelidinan M., Enayati A. (2025). Dengue Fever Resurgence in Iran: An Integrative Review of Causative Factors and Control Strategies. Trop. Mede Infect. Dis..

[78] Sedaghat M.M. (2025). Commentary: Dengue fever: A decade of burden in Iran. Front. Public Health.

[79] Humphrey J.M., Cleton N.B., Reusken C.B., Glesby M.J., Koopmans M.P., Abu-Raddad L.J. (2016). Dengue in the Middle East and North Africa: A systematic review. PLoS Negl. Trop. Dis..

[80] Vanslembrouck A., Jansen S., De Witte J., Janssens C., Vereecken S., Helms M., Lange U., Lühken R., Schmidt-Chanasit J., Heitmann A. (2024). Larval Competition between *Aedes* and *Culex* mosquitoes carries over to higher arboviral infection during their adult stage. Viruses.

