# Supplementary material for: Spatiotemporal Patterns and Historical Overview of Aedes Mosquitoes in Iran: A Systematic Review
Source: Trop Med Infect Dis. 2026 May 12;11(5):131. doi: 10.3390/tropicalmed11050131 (PMC13211443; doi:10.3390/tropicalmed11050131)
Supplement: Supplementary file 1 [file tropicalmed-11-00131-s001.zip › File S1 .pdf]

Electronic Search Strategy:

**The PubMed database was searched using the following strategy:**

((“Aedes”[Mesh] OR Aedes[tiab])
AND
(“Mosquitoes”[Mesh] OR “Culicidae”[Mesh] OR mosquito*[tiab])
AND
(Iran[tiab])).

Filters were applied to restrict the results to studies published between January 1980 and October 2025.

In addition, keywords such as “distribution”, “presence”, “emergence”, “surveillance”, and “monitoring” were considered during the screening and selection process to ensure comprehensive coverage of relevant studies.

Equivalent keyword-based searches were conducted in Scopus, Web of Science, ScienceDirect, Google Scholar, and Iranian databases using database-specific syntax adapted from the PubMed strategy.

**Scopus:**

(TITLE-ABS-KEY(aedes) AND TITLE-ABS-KEY(mosquito* OR culicidae) AND TITLE-ABS-KEY(Iran))

**Google Scholar:**

“Aedes” AND Iran AND (mosquito OR Culicidae)
