# Supplementary material for: Spatiotemporal Patterns and Historical Overview of Aedes Mosquitoes in Iran: A Systematic Review
Source: Trop Med Infect Dis. 2026 May 12;11(5):131. doi: 10.3390/tropicalmed11050131 (PMC13211443; doi:10.3390/tropicalmed11050131)
Supplement: Supplementary file 1 [file tropicalmed-11-00131-s001.zip › Table S1 .pdf]

Supplementary Table S1. Detailed entomological survey data on the spatial and temporal distribution of *Aedes* mosquitoes in Iran.

Reported *Aedes* species	Location	Total Mosquito Sample Size	Larval/adults sampling	Year	Ref. No.
Ae. vexans, Ae. geniculatus, Ae. caballus, Ae. caspius, Ae pulchritarsis, Ae. vittatus, Ae. echinus, Ae. detritus, Ae. flavescens, and Ae. leucomelas	147 districts of Iran	Not reported (2362 breeding places)	Both	1984	[34]
Ae. caballus in Sistan and Baluchestan, Hormozgan<br>*Ae. caspius* in Guilan, West Azerbaijan,<br>Zanjan, Lorestan, Khorasan, Isfahan, Chahar-Mahall, Yazd, Kerman, Sistan and Baluchestan, Hormozgan, Fars, Bushehr, Khuzestan<br>*Ae. detritus* in Hormozgan<br>*Ae. echinus* in Mazandaran<br>*Ae. flavescens* in West Azerbaijan<br>*Ae. geniculatus* in Guilan, Mazandaran<br>*Ae. leucomelas* in Hormozgan<br>*Ae pulchritarsis* in Mazandaran<br>*Ae. vexans* in Guilan, Mazandaran, West Azerbaijan, and Hormozgan<br>*Ae. vittatus* in Hormozgan	Most provinces nationwide	Not reported	Both	1987	[35]
Ae.caspius, Ae.echinus, Ae.geniculatus, Ae.vexans	Guilan Province	9134	Both (2478 adult, 6656 larvae)	2002	[37]
Ae. vexans, Ae. caspius	East Azerbaijan Province	1638	Both (1035 adult, 603 larvae)	2007	[38]
Ae. vexans, Ae. caballus, Ae. caspius	Chabahar county, Sistan and Baluchestan province	8855	Both (3824 adult, 5031 larvae)	2009	[29]
Ae. caspius, Ae. geniculatus, Ae. vexans	Ardabil Province	20623	Both (17533 adult, 3090 larvae)	2009	[68]
Ae. vexans, Ae. caspius	Sanandaj county, Kurdistan province	5647	Both (1862 adult, 3785 larvae)	2010	[39]
Ae. caspius	Iranian islands (Abu-Musa, Hormuz, Larak	521	Both	2010	[40]

Reported *Aedes* species	Location	Total Mosquito Sample Size	Larval/adults sampling	Year	Ref. No.
	and Qeshm), Hormozgan Province				
Ae. vexans, Ae. geniculatus, and Ae. echinus	Guilan province	6656	6656 larvae	2011	[64]
Ae. caspius	North Khorasan Province	1336	Both (682 adult, 654 larvae)	2011	[41]
Ae. caspius	Shadegan wetland in Khuzestan Province	2664	2664 adult	2012	[42]
Ae. caspius	Qom Province	371	371 larvae	2012	[43]
Ae. caspius	Kurdistan Province	2096	2096 larvae	2013	[44]
Ae. vittatus, Ae. caspius	Shadegan wetland in Khuzestan Province	1071	1071 larvae	2014	[45]
Ae. geniculatus, Ae. caspius	West Azerbaijan Province	1569	Both (233 adult, 1336 larvae)	2014	[46]
Ae. vexans, Ae. caspius	Kurdistan and Kermanshah	6094	Both (2013 adult, 4081 larvae)	2015	[47]
Ae. geniculatus	Neka County, Mazandaran Province	32530	32530 larvae	2015	[66]
Ae. caspius	Mahshahr district in Khuzestan Province	2641	Both (1023 adult, 1618 larvae)	2015	[48]
Ae. vexans, Ae. caspius	Isfahan Province	1659	1659 larvae	2015	[49]
Ae. caspius	West Azerbaijan Province	26	3 adult	2016	[50]
Ae. albopictus, Ae. caspius, Ae. caballus, Ae. flavescens, Ae. vexans in Sistan & Baluchestan<br>*Ae. caballus, Ae. vexans,* Ae. *vittatus* in Hormozgan<br>*Ae. caspius, Ae. vexans, Ae. detritus* in Bushehr<br>*Ae. caspius* in Khuzestan<br>*Ae. vexans* in Khorasan Jonobi<br>*Ae. caspius* in Kerman<br>No *Aedes* mosquitoes were collected from Fars and Ilam Provinces.	Eight provinces (Sistan and Baluchestan, Hormozgan, Bushehr, Khuzestan, Khorasan Jonobi, Kerman, Fars and Ilam	11920	Both (3734 adult, 8186 larvae)	2016	[20]
Ae. vexans, Ae. caballus, Ae. caspius	Bashagard County, Hormozgan Province	1479	1479 larvae	2017	[30]
No *Aedes* mosquitoes were collected	Firouzabad County, Fars	2002	Both (689 adult, 1313 larvae)	2017	[11]
No *Aedes* mosquitoes were collected	Mazandaran Province	19840	19840larvae	2017	[12]

Reported *Aedes* species	Location	Total Mosquito Sample Size	Larval/adults sampling	Year	Ref. No.
Ae. caspius, Ae. vexans, Ae. detritus, Ae. albopictus, and *Ae. unilineatus*.	Sistan and Baluchestan Province	1885	1885 adult	2017	[25]
Ae. caspius, Ae. echinus, Ae. geniculatus	Kalaleh County, Golestan Province	727	Both (332 adult, 395 larvae)	2017	[51]
Ae. caspius	Golestan Province	2891	2891 larvae	2018	[52]
Ae. caspius, Ae. vexans	Mazandaran Province	36024	Both (16184 adult, 19840 larvae)	2018	[53]
Ae. caspius	Sistan and Baluchestan, Hormozgan, Bushehr and Khuzestan	1769	1769 adult	2018	[54]
Ae. caspius	Golestan Province	5661	5661 larvae	2018	[55]
Ae. caspius, Ae. vexans, Ae. flavescens	Ardabil Province	694	694 larvae	2018	[56]
Ae. caspius, Ae. vexans, Ae. geniculatus, Ae. pulcritarsis	Guilan Province	16327	16327 adults	2018	[67]
Ae. caspius, Ae. flavescens, Ae. vexans	Ardabil Province	2087	2087 larvae	2018	[57]
Ae. vexans	Guilan Province	1015	1015 adults	2019	[69]
Ae. caspius, Ae. vexans	East Azerbaijan Province	1401	Both (10165 adult, 386 larvae)	2019	[58]
No *Aedes* mosquitoes were collected	Lorestan province	4211	4211 adults	2020	[13]
Ae. caspius, Ae. vexans, Ae. caballus	Sistan and Baluchestan	4878	4878 adults	2020	[31]
Ae. caspius	West Azerbaijan Province	2715	2715 larvae	2020	[59]
Ae. caspius	Hormozgan Province	….	Both adult, larvae	2020	[60]
No *Aedes* mosquitoes were collected	Lenjan and Mobarakeh Counties, Isfahan Province	430	430 larvae	2021	[14]
No *Aedes* mosquitoes were collected	Karzin from Fars province	1884	1884 larvae	2021	[15]
Aedes aegypti	Hormozgan Province	8080	Both (4560 adult, 3520 larvae)	2021	[21]
Ae. caspius, Ae. vexans, Ae. flavescens, Ae. caballus, Ae. albopictus	Eight provinces (Sistan & Baluchestan, Hormozgan, Bushehr, Khuzestan, Khorasan Jonobi, Kerman, Fars and Ilam	8188	8188 larvae	2021	[26]

Reported *Aedes* species	Location	Total Mosquito Sample Size	Larval/adults sampling	Year	Ref. No.
No *Aedes* mosquitoes were collected	Sanandaj County, Kurdistan Province	1543	Both (342 adult, 1201 larvae)	2021	[16]
No *Aedes* mosquitoes were collected	Lorestan province	4805	Both (4363 adult, 442 larvae)	2021	[17]
No *Aedes* mosquitoes were collected	Kashan County, Isfahan Province	9789	9789 larvae	2021	[18]
Ae vexans, Ae geniculatus, Ae. echinus, Ae. pulchritarsis	Guilan Province	3964	Both adult and larvae	2022	[65]
Ae. caballus	Hormozgan Province	5137	Both (1901 adult, 3236 larvae)	2022	[32]
No *Aedes* mosquitoes were collected	Kurdistan province	5831	5831 larvae	2022	[19]
Ae. aegypti, Ae. caspius, Ae. vexans, Ae. caballus, Ae. vittatus	Hormozgan Province	16716	Both (4843 adult, 11873 larvae)	2022	[22]
Ae. vexans, Ae. geniculatus, Ae. caspius, Ae. echinus, Ae. pulcritarsis, Ae. favescence	Mazandaran Province	4410	Both (2034 adult, 2376 larvae)	2023	[7]
Ae. vittatus, Ae. leucomelas, Ae. detritus, Ae. favescence	Sirjan County, Kerman Province	4538	4538 larvae	2023	[63]
Ae. caspius, Ae. vexans	Hormozgan Province	6165	6165 adults	2023	[61]
Ae. aegypti, Ae. caspius, Ae. vexans, Ae. caballus, Ae. vittatus	Hormozgan Province	1351	Both (452 adult, 899 larvae)	2023	[23]
Ae. aegypti	Bandar Lengeh in Hormozgan Province	45	Both (5 adult, 40 larvae)	2024	[24]
Ae. caballus	Chabahar and Kanarak, Sistan and Baluchestan Province	1734	1734 adults	2024	[33]
Ae. albopictus	Guilan Province	925	Both (896 adult, 29 larvae)	2024	[28]
Ae. cinereus	Guilan Province	134	134 adults	2024	[36]
Ae. aegypti	Bandar Abbas City, Hormozgan Province	30	1465 eggs	2024	[8]
Ae. caspius	Qom Province	83414	83414 adults	2025	[62]
Ae. albopictus, Ae. caspius, Ae. vexans	Ardabil Province	7670	Both (206 adult, 7464 larvae)	2025	[27]
